# Correlation Between Gait and Near-Infrared Brain Functional Connectivity Under Cognitive Tasks in Elderly Subjects With Mild Cognitive Impairment

**DOI:** 10.3389/fnagi.2021.482447

**Published:** 2021-06-11

**Authors:** Ying Liu, Congcong Huo, Kuan Lu, Qianying Liu, Gongcheng Xu, Run Ji, Tengyu Zhang, Pan Shang, Zeping Lv, Zengyong Li

**Affiliations:** ^1^Beijing Key Laboratory of Rehabilitation Technical Aids for Old-Age Disability, National Research Center for Rehabilitation Technical Aids, Beijing, China; ^2^Rehabilitation Hospital, National Research Center for Rehabilitation Technical Aids, Beijing, China; ^3^Key Laboratory of Neuro-functional Information and Rehabilitation Engineering of The Ministry of Civil Affairs, Beijing, China; ^4^China Academy of Information and Communications Technology, Beijing, China; ^5^China Electronics Standardization Institute, Beijing, China

**Keywords:** mild cognitive impairment, functional connectivity, wavelet phase coherence, gait symmetry, dual-tasks

## Abstract

Older adults with mild cognitive impairment (MCI) have a high risk of developing Alzheimer’s disease. Gait performance is a potential clinical marker for the progression of MCI into dementia. However, the relationship between gait and brain functional connectivity (FC) in older adults with MCI remains unclear. Forty-five subjects [MCI group, *n* = 23; healthy control (HC) group, *n* = 22] were recruited. Each subject performed a walking task (Task 01), counting backward–walking task (Task 02), naming animals–walking task (Task 03), and calculating–walking task (Task 04). The gait parameters and cerebral oxygenation signals from the left prefrontal cortex (LPFC), right prefrontal cortex (RPFC), left motor cortex (LMC), right motor cortex (RMC), left occipital leaf cortex (LOL), and right occipital leaf cortex (ROL) were obtained simultaneously. Wavelet phase coherence was calculated in two frequency intervals: low frequency (interval I, 0.052–0.145 Hz) and very low frequency (interval II, 0.021–0.052 Hz). Results showed that the FC of RPFC–RMC is significantly lower in interval I in Task 03 compared with that in Task 02 in the MCI group (*p* = 0.001). Also, the right relative symmetry index (IDpsR) is significantly lower in Task 03 compared with that in Task 02 (*p* = 0.000). The IDpsR is positively correlated with the FC of RPFC–RMC in interval I in the MCI group (*R* = 0.205, *p* = 0.041). The gait symmetry such as left relative symmetry index (IDpsL) and IDpsR is significantly lower in the dual-task (DT) situation compared with the single task in the two groups (*p* < 0.05). The results suggested that the IDpsR might reflect abnormal change in FC of RPFC–RMC in interval I in the MCI population during Task 03. The gait symmetry is affected by DTs in both groups. The findings of this study may have a pivotal role in the early monitoring and intervention of brain dysfunction among older adults with MCI.

## Introduction

Mild cognitive impairment (MCI) is a transitional state between normal aging and early dementia. The annual conversion rate of MCI into Alzheimer’s disease is 8–15%, and MCI is the appropriate stage for preventive intervention (Prince et al., 2016). However, the population base is large, the community lacks doctors with diagnostic capabilities, the examination methods are cumbersome, and the patient compliance requirements are high. The early screening and diagnosis of MCI are important (Barry and Serge, 2008), but early objective evaluation indicators for MCI are lacking.

Gait refers to the postural and behavioral characteristics of the human body while walking. Gait parameters are commonly used to assess the risk for MCI in a population. These parameters assess the process of moving the body in a certain direction by measuring a series of continuous activities of the hip, knee, ankle, and toe (Montero-Odasso et al., 2012). Gait analysis is used to reveal the gait abnormalities based on biomechanics and kinematics in subjects with MCI during walking (Crockett et al., 2017). Abnormal gait is a prevalent feature among older adults with cognitive impairment (Ganz et al., 2007). Abnormal gait in the elderly can be attributed to neurologic diseases, arthritis, and acquired foot deformities (Alexander, 1996; Verghese et al., 2002; Wilson et al., 2002).

Dual-task (DT) gait testing is used to assess the interaction among cognition, gait, and fall risk. The DT paradigm refers to the observation of people walking while performing a second task that needs attention and reflects the relationship between cognition and gait (Tian et al., 2017). Gait variability is correlated with fall risk, disease duration and severity, and motor and cognitive functions. Increased gait variability and decreased walking speed are demonstrated in subjects with MCI during the DT test with increasing DT complexity (Tian et al., 2017). It has been demonstrated that abnormal brain regions were related to motor function in subjects with Parkinson’s disease under resting state (Canu et al., 2016).

More challenging mobility performance tasks, such as DT walking, may better capture early brain abnormalities (Beauchet et al., 2013). For example, the hippocampus (H) has a functional connection with the prefrontal cortex (PFC) through the entorhinal cortex (E) and the substantia nigra striatum (NS) system (Jordan et al., 2004). The degradation of the hippocampus leads to the decomposition of visual, vestibular, and proprioceptive sensory and contextual information into spatial maps, leading to gait disorders. Damage to the PFC may lead to executive dysfunction, leading to gait disturbance (Jordan et al., 2004).

Functional near-infrared spectroscopy (fNIRS) is non-invasive, secure, and cheap, and it exhibits good mobility and high time resolution (Bernjak et al., 2012; Ferrari and Quaresima, 2012). fNIRS maps human brain functions by measuring local changes in the hemoglobin concentrations in the brain (Boas et al., 2014; Scholkmann et al., 2014). Functional connectivity (FC) can be derived from fNIRS signals. FC reflects the important connections among different spatial regions of the cerebral cortex. The FC network can reveal the intrinsic characteristics of the brain network (Biswal et al., 1995; Fox and Raichle, 2007). Studies on FC based on fNIRS have been popular in recent years (Fox and Raichle, 2007). Some studies have used fNIRS to evaluate the prefrontal brain activation of healthy young people in the walking environment of working memory tasks. These studies found that in young people, the neural related factors of executive function and dynamic posture control tend to be concurrent with the walking environment and process. The cognitive load changes with different tasks (Lin and Lin, 2016). However, the specific relationship between gait and FC in older adults with MCI while performing tasks remains unclear.

The hypothesis of this study is that gait abnormalities are related to brain function in subjects with MCI. This study aims to (1) analyze the brain FC of older adults with MCI during the single task and DTs and (2) examine the correlation between brain FC and gait symmetry indices during the single task and DTs based on the gait and cerebral oxygen parameters recorded simultaneously using the Vicon three-dimensional (3D) dynamic capture system and portable fNIRS device.

## Materials and Methods

### Subjects

In this study, 45 elderly subjects were recruited from a local community. The inclusion criteria for patients were as follows: (a) no abnormal brain structure, such as contusions caused by tumors and head trauma, which may impair cognitive function; (b) objective evidence of impairment in the cognitive domain: memory, executive function/attention, language, or vision spatial skills; (c) normal functional activities; (d) no dementia (Park et al., 2011). After screening, the MCI and healthy control (HC) groups were formed with 20 and 17 members, respectively. The experimental procedure was approved by the Ethics Committee of the National Research Center for Rehabilitation Technical Aids. Informed consent was obtained from each participant before the experiment. Table 1 shows the information regarding the age, body mass index, blood pressure, and Mini-Mental State Examination (MMSE) and Montreal Cognitive Assessment (MoCA) scores of the subjects before the experiment.

**TABLE 1:** Basic information of the subjects.

Parameters	MCI group (standard deviations)	HC group (standard deviations)	*t*	*p*
			
			Independent sample *T*-test	
Age (years)	62.41 (5.03)	64.38 (5.46)	0.914	0.389
Body mass index (BMI)	24.27 (2.51)	25.23 (2.43)	0.639	0.533
Systolic blood pressure (mmHg)	69.10 (4.38)	74.81 (4.68)	−2.571	0.039
Diastolic blood pressure (mmHg)	128.26 (3.54)	112.83 (5.34)	−1.064	0.316
Mini-Mental State Examination (MMSE)	27.64(1.76)	29.09 (1.15)	−1.738	0.122
Montreal Cognitive Assessment (MoCA)	22.64 (2.54)	27.05(1.29)	−5.522	0.000

### Gait Test

The subjects wore the same tight shorts and sneakers. Sixteen marks were pasted strictly in accordance with Plug-in Gait requirements (Kadaba et al., 1990). The motion capture system using 12 cameras (Vicon, Oxford Metrics Limited, United Kingdom) was used to collect kinematic data at a sampling frequency of 100 Hz. Two force platforms (OR-65, AMTI, United States) were used to record ground reaction force (GRF) data at a sampling frequency of 1.5 kHz. At the beginning of the experiment, the subjects walked back and forth on a fixed path.

### Mental Tasks

During the test, the MMSE and MoCA scales were used to assess the cognitive level of the subjects. The evaluation and diagnosis of MCI and HC groups were completed by doctors from the Affiliated Hospital of the National Rehabilitation Aid Research Center. The assessment scale was combined with the main complaints and clinical history information and recognition provided by patients and their families in the evaluation process to determine the sample grouping. Patients or families of MCI subjects had subjective cognitive decline complaints. The MMSE scores were between 24 and 29 (Stokholm et al., 2006), and the MoCA scores of all subjects were between 22 and 26 (Freitas et al., 2012). All subjects had lower memory and computational performance in MMSE and MoCA scales. The scores of the MMSE and MoCA scales of the HC group must be greater than the prescribed normal scores.

### Functional Near-Infrared Spectroscopy Measurements

**FIGURE 1:**
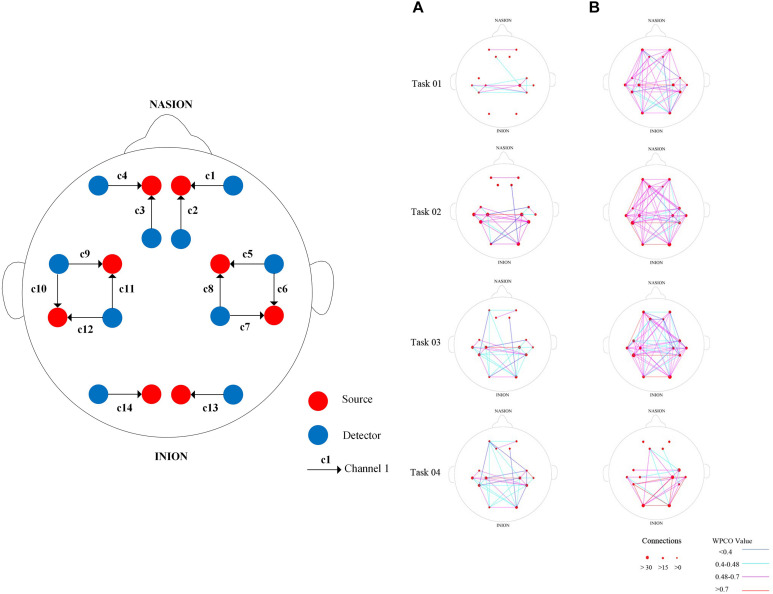
Near-infrared channel probe layout **(A)** and typical examples of diagram of brain functional connectivity (FC) in interval I from the two groups. Arrangement of source optodes (red dots, S), detector optodes (blue dots, D), and measurement channels (black arrow, C) in the prefrontal cortex, motor cortex, and occipital leaf cortical areas based on the international 10/10 system. **(B)** Comparisons of wavelet phase coherence (WPCO) in the 15 connectivity types among the groups. (a) WPCO of MCI group in four states of interval I. (b) WPCO of control group in four states of interval I. Red lines represent WPCO values greater than 0.7. Pink lines represent WPCO values between 0.48 and 0.7. Light green lines represent WPCO values between 0.4 and 0.48. Blue lines represent WPCO values under 0.4. The size of the red dot at channel position represents the density of connection on this channel. The largest red dot means the number of connections on this channel is greater than 30; medium red dot, the number is between 0 and 15; smallest red dot, the number is 0–14. a, MCI group; b, healthy control (HC) group.

A 14-channel fNIRS device (Danyang Huichuang Medical Equipment Co., Ltd.) was used to collect cerebral blood oxygen signals, and the brain region position used for the measurements was based on the internationally used 10/10 electrode position (Oostenveld and Praamstra, 2001). Light sources and detectors were placed in the following cortical areas: left prefrontal cortex (LPFC), right prefrontal cortex (RPFC), left motor cortex (LMC), right motor cortex (RMC), left occipital leaf cortex (LOL), and right occipital leaf cortex (ROL). The distance between the source and detector was 30 mm. The sampling frequency was set at 10 Hz. Figure 1A shows the arrangement of the probe for the measurement and typical examples of the diagram of brain FC. A light source and a detector formed the channel.

### Areas Tested by Functional Near-Infrared Spectroscopy

#### Prefrontal Cortex (PFC)

It is generally considered to be the human cognitive cortical area (Yang et al., 2001; Wang et al., 2005). This cortical area participates in the execution process, including working memory, attention resource allocation, and plot information processing, especially working memory in a DT context (Gouwanda, 2014). Therefore, PFC is a suitable research object to investigate changes in brain activity related to distraction.

#### Motor Cortex (MC)

It participates in feeling the posture and movement of the human body and controls the contralateral limb. Movement plays an important role in people’s feelings and movement control.

#### Occipital Leaf (OL)

It is mainly used for visual information processing and is also associated with functions such as memory and motor perception (Wu and Pan, 2014). Figure 1B shows the arrangement of the probe for the measurement and typical examples of the diagram of brain FC. A light source and a detector formed the channel.

### Experimental Procedure

**FIGURE 2:**
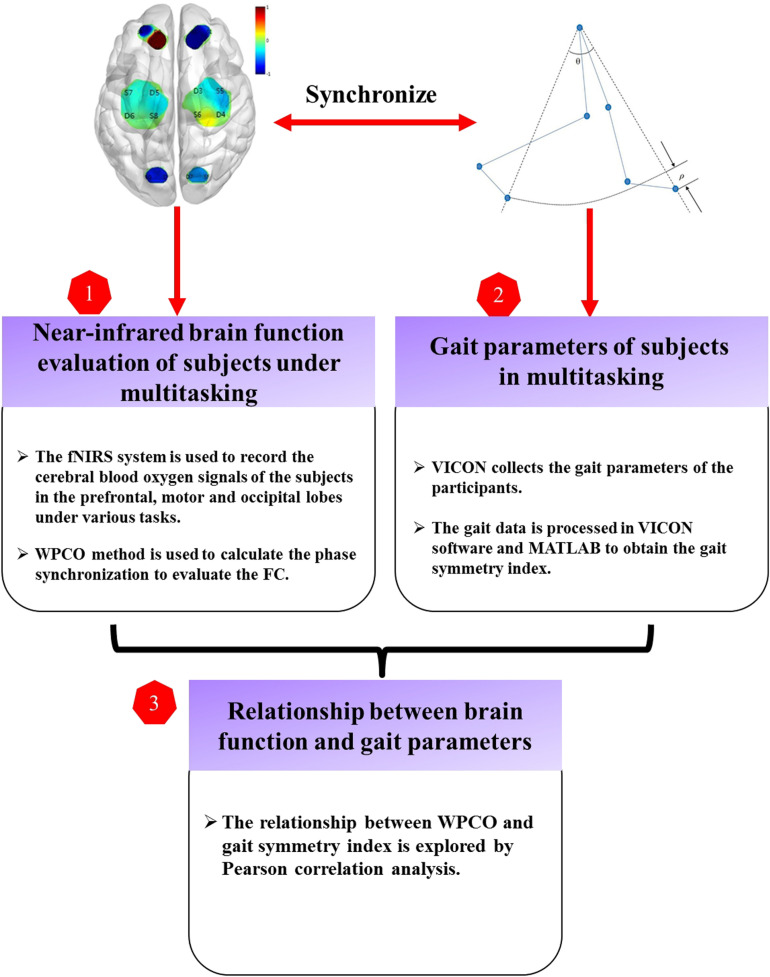
Technology road map. FC, functional connectivity; fNIRS, functional near-infrared spectroscopy; WPCO, wavelet phase coherence.

Three-dimensional gait analysis was performed in a gait laboratory. The cerebral blood oxygen signals were obtained using a portable fNIRS instrument (Danyang Huichuang Medical Equipment Co., Ltd.). Each subject performed four different tasks: walking task (Task 01), counting backward–walking task (Task 02), naming animals–walking task (Task 03), and calculating–walking task (Task 04) (Hittmair-Delazer et al., 1994; Weiss et al., 2003). Task 01 was a single task, whereas Task 02, Task 03, and Task 04 were DTs. The subjects were asked to walk for 10 min in a comfortable manner on a 3-m walkway in each task. A 5-min break was provided between experiments to prevent fatigue. Each task was demonstrated to each participant to ensure that he or she was familiar with the experimental process before the experiment began. The laboratory provided the subjects’ experimental clothes with uniform lower body and disposable shorts to facilitate labeling. The research idea and scheme of this study are shown in Figure 2.

### Gait Analysis

#### 3D Motion

Before each gait test, the system should be calibrated. Marks were pasted on the subjects (according to Plug-in Gait requirements) to collect gait actions. A new database on Vicon Nexus was created, and then a static model was established to collect gait actions.

#### Data Processing

**FIGURE 3:**
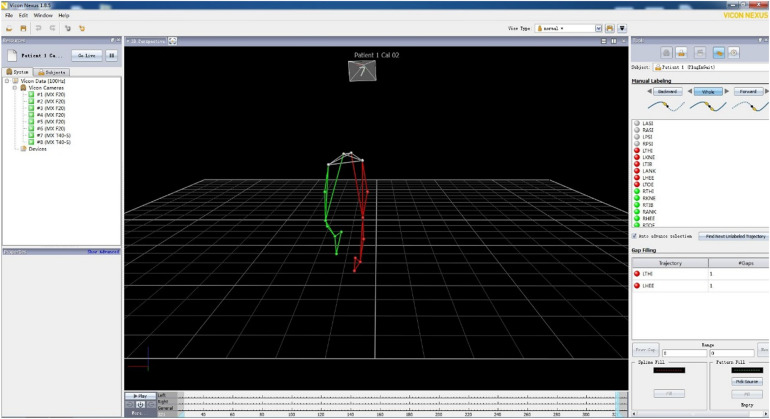
Processing gait data in VICON.

**FIGURE 4:**
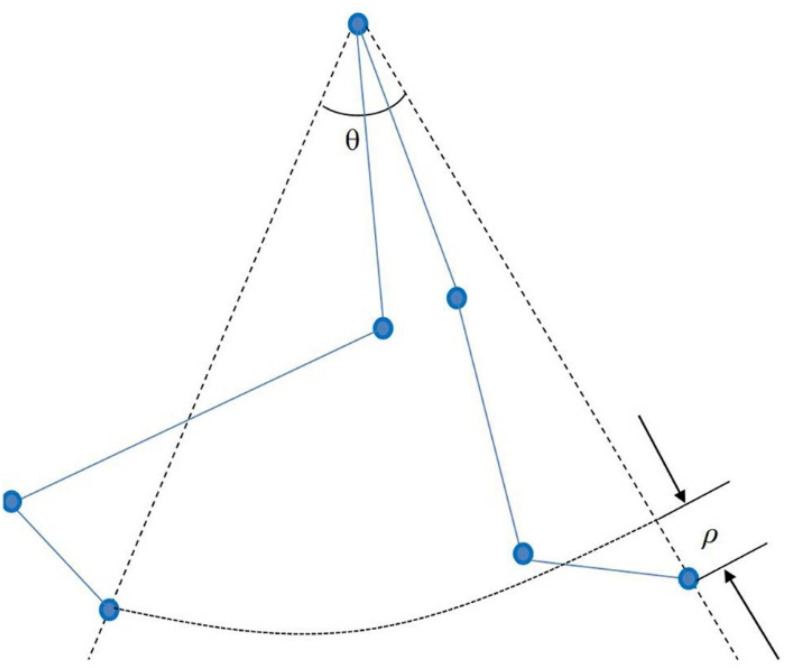
Definition of lower limb gait characteristics in polar coordinates.

The start and end time of the video were set for analysis, and the marked points were identified and checked. The processed image data could be viewed and output through the software. Finally, the output processing data were imported into the Matlab software gait parameter script to run to complete the calculation of gait symmetry parameters, including gait speed, step length, step length change, DT speed consumption, and other parameters. Figure 3 shows the processing gait data in VICON. A prominent feature of the normal gait of the human body is the symmetry of motion. The effects of special or abnormal conditions (e.g., crossing obstacles and walking dysfunction) on the overall characteristics of gait hinder the symmetry of walking. Therefore, the symmetry of gait is important for the comprehensive evaluation of gait movement and walking function (Wu and Pan, 2014). See the Appendix for the calculation formula of gait symmetry indices. Figure 4 shows the definitions of lower limb gait features in polar coordinates.

### Wavelet Phase Coherence

Wavelet phase coherence (WPCO) is a brain FC analysis method for illustrating the phase relationship of brain function adjustment (Bernjak et al., 2012). A large WPCO value indicates a high phase synchronism and strong FC. In this study, the phase synchronization value was calculated using the WPCO method and used in the analysis of the FC of each brain region. The WPCO was calculated in two frequency intervals: interval I was at 0.052–0.145 Hz, and interval II was at 0.021–0.052 Hz. See the Appendix for the formula of WPCO.

Amplitude-adjusted Fourier transform (AAFT) was used to analyze the phase synchronization between cerebral blood oxygen signals to test its true level. A total of 100 AAFT replacement signals were calculated for each blood oxygen signal, and the 100 phase coherence values between the corresponding substitute signals of each of the two blood oxygen signals were calculated. The WPCO value between the two blood oxygen signals was considered significant if the measured WPCO value was greater than the mean of the corresponding 100 substitute signal WPCO values plus twice the standard deviation (Gao et al., 2015). Otherwise, the WPCO value was not significant, and no FC existed between the corresponding channels (Theiler et al., 1992).

The WPCO between every two channels was calculated using the WPCO analysis method. The trapezoidal integral was divided by the band range to obtain the WPCO mean between the two channels. Fifteen functional zone pairs, namely, LPFC–RPFC, LPFC–LMC, LPFC–RMC, LPFC–LOL, LPFC–ROL, RPFC–LMC, RPFC–RMC, RPFC–LOL, RPFC–ROL, LMC–RMC, LMC–LOL, LMC–ROL, RMC–LOL, RMC–ROL, and LOL–ROL, were formed from the six cortical regions.

## Statistical Analysis

The Shapiro–Wilk’s test and Levene’s test were used to test the normal distribution and homogeneity of variance of the two groups (Wu and Pan, 2014). For the data with non-normal distribution or uneven variance, the non-parametric method Mann–Whitney *U* test was used to compare the two sets of data. For the normal distribution and homogeneity of variance, the parametric method *T*-test was used. The effects of different conditions on FC and gait were performed using the repeated ANOVA. Correction for multiple comparisons was performed with Bonferroni method. The Pearson correlation coefficient was used to analyze the correlation between the WPCO value and the gait symmetry index, and the *p*-value of Pearson’s correlation coefficient was less than 0.05 (Wu and Pan, 2014).

## Results

### Comparison in FC Among the Four Tasks

**FIGURE 5:**
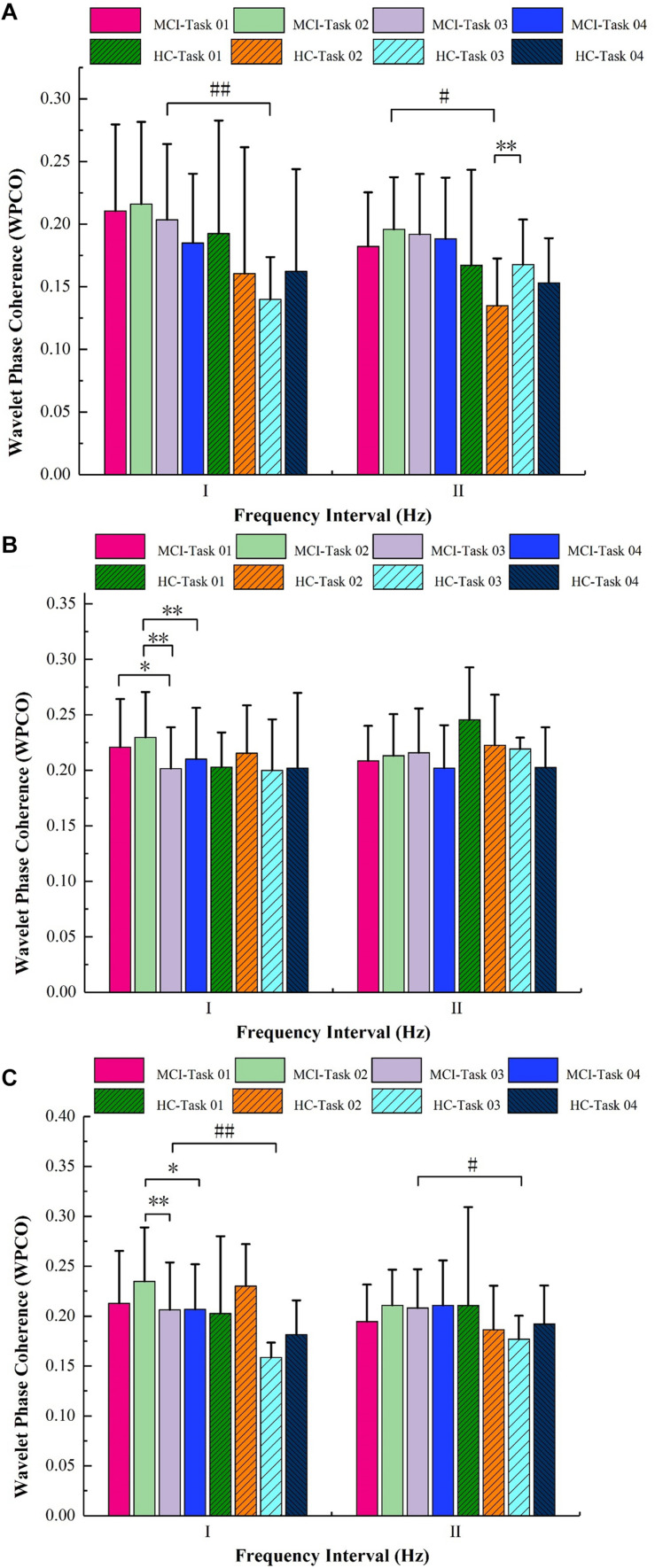
Results of wavelet phase coherence (WPCO) in different brain regions among the groups under the four tasks. **(A)** WPCO of left prefrontal cortex–right occipital leaf cortex (LPFC–ROL), **(B)** WPCO of right prefrontal cortex–right motor cortex (RPFC–RMC), **(C)** WPCO of left prefrontal cortex–left motor cortex (LPFC–LMC). Significant levels are marked with ^#^*p* < 0.05, ^##^*p* < 0.01, ^∗^*p* < 0.05, ^∗∗^*p* < 0.01. HC, healthy control; MCI, mild cognitive impairment.

Figure 5 shows the comparisons in WPCO in interval I and interval II. The results show that the FC of RPFC–RMC in the MCI group is significantly lower in interval I in Task 03 compared with in Task 02 (*p* = 0.001). The FC in interval I is significantly higher in the MCI group than that in the HC group (*p* = 0.008) in Task 03. The FC in interval II is significantly higher in MCI group than that in HC group (*p* = 0.017) in Task 02.

The repeated ANOVA showed that the FCs of LPFC–ROL were significantly different in interval II in the HC group among the four tasks (*p* < 0.05). The FC of LPFC–ROL in interval II is significantly higher in Task 03 compared with that in Task 02 (*p* = 0.000).

### Comparison in Gait Parameters Among the Four Tasks

**FIGURE 6:**
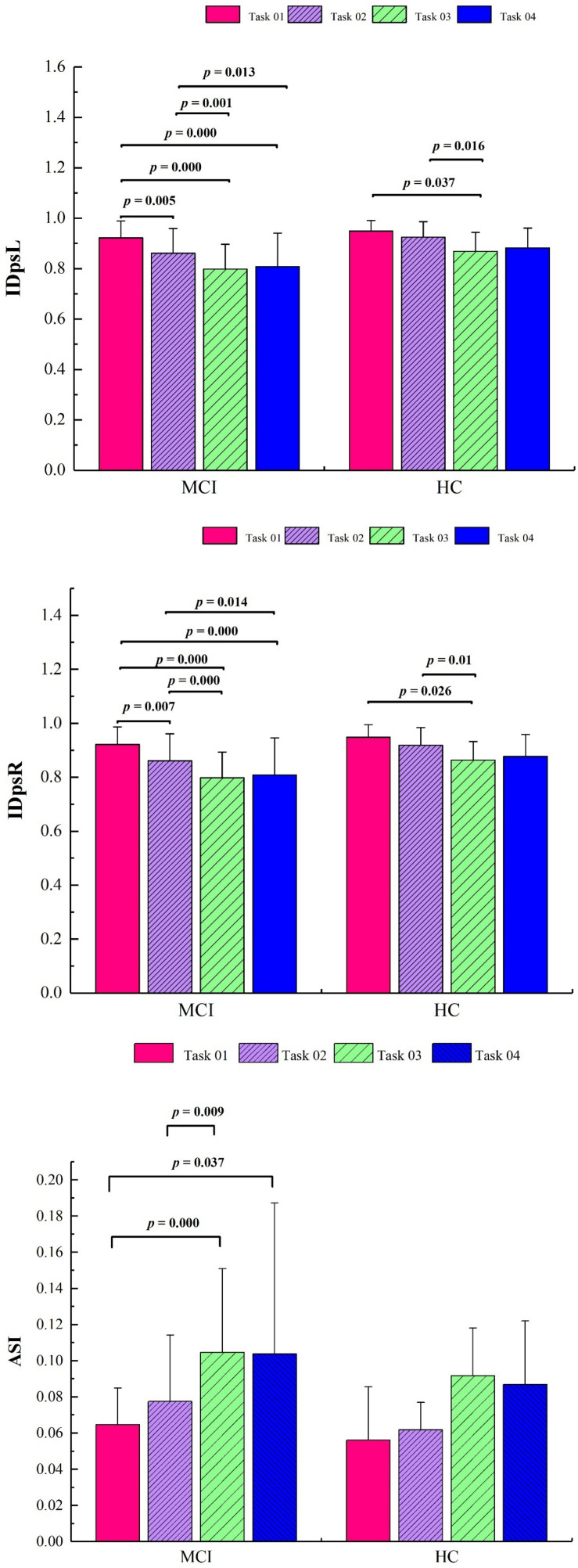
Significant results of gait symmetry indices for four tasks in the two groups. IDpsL, left relative symmetry index; IDpsR, right relative symmetry index; ASI, asymmetry index; HC, healthy control; MCI, mild cognitive impairment.

As shown in Figure 6, the right relative symmetry index (IDpsR) was significantly lower in Task 03 compared with Task 02 in the MCI group (*p* = 0.000). The asymmetry index (ASI) values were significantly higher in Task 03 (*p* = 0.000) and Task 04 (*p* = 0.037) compared with those in Task 01. Compared with that of Task 02, the ASI value was significantly higher in Task 03 (*p* = 0.009). Calculated using repeated measurements of ANOVA, the ASI was significantly different in the MCI group (*p* < 0.05).

### Correlation Between Functional Connectivity and Gait Parameters

**FIGURE 7:**
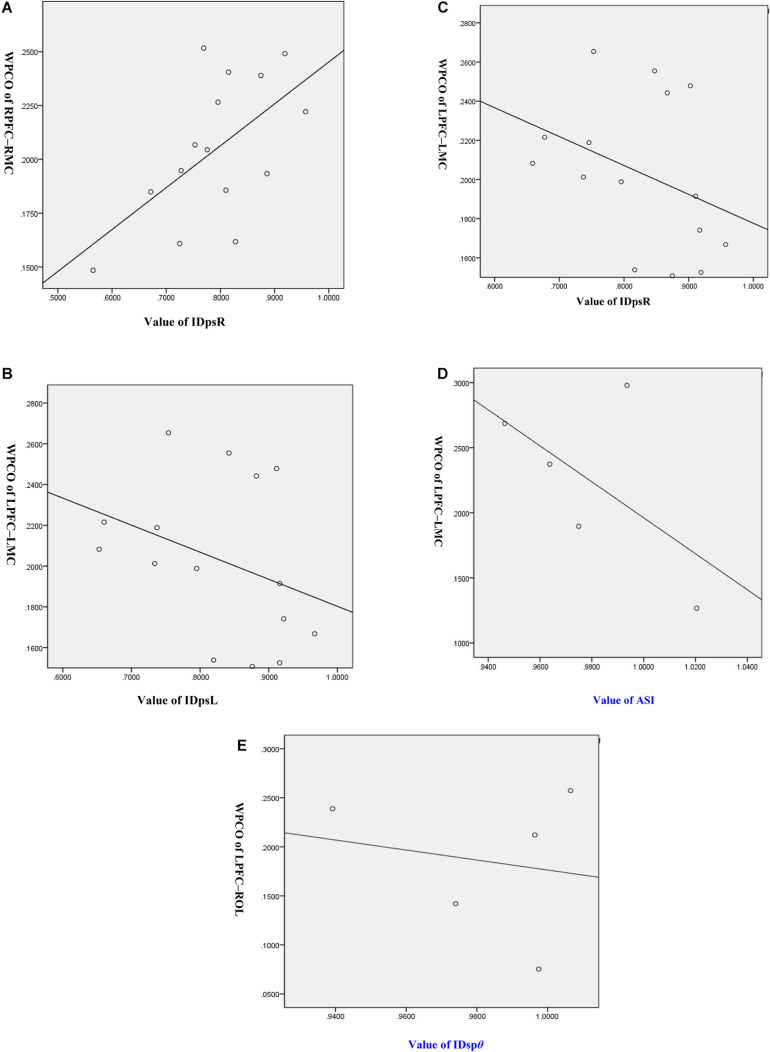
Correlation analysis of wavelet phase coherence (WPCO) values and gait symmetry values. **(A)** Correlation analysis between WPCO values of RPFC-RMC and values of IDpsR. **(B)** Correlation analysis between WPCO values of LPFC-LMC and values of IDpsL. **(C)** Correlation analysis between WPCO values of LPFC-LMC and values of IDpsR. **(D)** Correlation analysis between WPCO values of LPFC-LMC and values of ASI. **(E)** Correlation analysis between WPCO values of LPFC-ROL and values of IDspθ. IDpsL, left relative symmetry index; IDpsR, right relative symmetry index; ASI, asymmetry index; IDspθ, the step size symmetry index in polar coordinates; RPFC, right prefrontal cortex; LPFC, left prefrontal cortex; RMC, right motor cortex; LMC, left motor cortex; ROL, right occipital leaf cortex.

Figures 7A–E show the results of the Pearson correlation analysis between FC and gait parameters. In the MCI group, the IDpsR is positively correlated with the FC of RPFC–RMC in interval I (*R* = 0.205, *p* = 0.041). However, the relative symmetry index (IDpsL and IDpsR) is negatively correlated with the FC of LPFC–LMC in interval II group (*R* = -0.234, *p* = 0.019; *R* = -0.225, *p* = 0.024). In the HC group, the ASI is positively correlated with the FC of LPFC–LMC in interval II (*R* = 0.472, *p* = 0.035). Also, the step size symmetry index in polar coordinates (IDps*θ*) is positively correlated with the FC of LPFC–ROL in the HC group (*R* = 0.457, *p* = 0.043).

## Discussion

The purpose of this study is to analyze the brain FC of older adults with MCI during the single task and DTs and examine the correlation between brain FC and gait symmetry indices during the single task and DTs. The main findings are as follows: (1) The gait symmetry indices show a remarkable decrease during DT compared with those during the single task in both groups. (2) The IDpsR could reflect the abnormal change in FC of RPFC–RMC in interval I in the MCI group during naming animals–walking task.

Prior studies have been demonstrated that more challenging mobility performance tasks, such as DT walking, may better capture early brain abnormalities (Camicioli et al., 1997; Beauchet et al., 2013). During the execution of DTs, the neural network of each single task does not operate independently or in parallel but integrates with other brain regions to form a neural network to further improve executive function (Dove et al., 2000). Walking and counting backward (Task 02) examines working memory and attention. Walking and naming animals (Task 03) are about verbal fluency and relies on semantic memory. Walking while calculating (Task 04) examines working memory and attention. Working memory, attention, episodic recall, and conscious perception extensively activate the frontal and parietal cortex. However, there are difficulties in locating the executive function brain areas in the DT processing and exploring their specific functions, and the functional neuroanatomy of the processing has not reached consensus (Dove et al., 2000).

In the present study, the FC of LPFC–ROL in interval II is significantly higher in the MCI group than that in the HC group in counting backward–walking task (Task 02). The hemodynamic parameters in interval II are closely regulated through tight neurovascular coupling and partial autonomic control within the brain (Zhang et al., 2002). The PFC is widely recognized as the cognitive cortical region of humans (Dove et al., 2000; Morgan et al., 2011; Schättin et al., 2016). The cortical area participates in the execution process, including working memory, attention resource allocation, and plot information processing, especially in the context of DTs (Pashler et al., 2001). The OL is mainly used for visual information processing and is also associated with functions such as memory and motor perception (Wu and Pan, 2014). The counting backward–walking task examines working memory and attention. Thus, the higher FC of LPFC–ROL in interval II might suggest that the MCI group needs more visual information processing to regulate the counting backward cognitive task than the HC group under the regulation of neural activity.

The frequency interval I is associated with changes in the peripheral sympathetic nerve activities that reflect sympathetically mediated and local myogenic mechanisms (Zhang et al., 2002). In the present study, the FC of RPFC–RMC in interval I is significantly lower in naming animals–walking task compared with that in counting backward–walking task in the MCI group. Counting backward–walking task examines working memory and attention. Naming animals–walking task is about verbal fluency and relies on semantic memory. The lower FC of RPFC–RMC suggests that naming animals–walking task relies less on executive function.

The PFC is considered an essential part of working memory. The PFC has a functional connection with the hippocampus (H) through the entorhinal cortex (E) and the substantia nigra striatum (NS) system. The degradation of the hippocampus leads to the decomposition of visual, vestibular, and proprioceptive sensory and contextual information into spatial maps and thus leading to gait disorders. Therefore, damage to the PFC may lead to executive dysfunction, leading to gait disturbance (Jordan et al., 2004; Tan et al., 2016).

Gait symmetry is an important aspect of the comprehensive evaluation of gait movement and walking function (Jin and Zhang, 2011). The values of IDpsL and IDpsR are between 0 and 1. A value closer to 1 indicates a better gait symmetry (Scholkmann et al., 2014). In the present study, the gait symmetry is reduced in the DT situation compared with the single task in the two groups. The results further confirm that gait symmetry is affected by cognitive tasks (Dove et al., 2000).

The IDps*θ* exhibits a positive correlation with the FC of LPFC–ROL in interval II in the HC group in Task 02. However, there was no significant correlation between FC and gait parameters in the MCI group. The results suggest that compared with the MCI group, the HC group has a stronger ability to adapt to cognitive tasks under the regulation of neural activity.

IDpsR is positively correlated with the FC of RPFC-RMC in the MCI group. At the same time, compared with counting backward–walking task, the IDpsR of naming animals–walking task decreased significantly in the MCI group. Therefore, the IDpsR might reflect abnormal change in FC of RPFC-RMC in interval I in the MCI population during naming animals–walking task under myogenic activities.

The hemodynamic parameters are closely regulated through tight neurovascular coupling and partial autonomic control in interval II within the brain (Zhang et al., 2002). In interval II, the FC of LPFC–LMC under naming animals–walking task of the MCI group is significantly higher than that of the HC group. At the same time, the FC of the MCI group at this connectivity is negatively correlated with IDpsL and IDpsR. However, the FC of the HC group at this connectivity is positively correlated with ASI. Therefore, naming animals–walking task, the FC was adjusted with different gait parameters to complete the task under tight neurovascular coupling and partial autonomic control.

### Limitations

The consideration of the results must further address the system activity interference within the set interval. In this study, interval I ranges from 0.052 to 0.145 Hz. The interference caused by heart rate and blood pressure fluctuations cannot be completely ruled out. The frequency interval I (0.06–0.15 Hz) is associated with changes in the peripheral sympathetic nerve activities that reflect sympathetically mediated and local myogenic mechanisms (Zhang et al., 2002). In one study, systemic signals contribute only 35% of hemodynamic changes when oxyhemoglobin is carried out in the frequency interval of 0.04–0.15 Hz (Katura et al., 2006). Therefore, these system activity disturbances may affect the results of FC, and the Meyer wave disturbances should be considered in future research.

## Conclusion

The IDpsR might reflect the abnormal change in FC of RPFC–RMC in interval I in the MCI group during the naming animals–walking task. The gait symmetry is affected by DTs in both groups. The findings of this study may have a pivotal role in the early monitoring and intervention of brain dysfunction among older adults with MCI.

## Data Availability Statement

The raw data supporting the conclusions of this manuscript will be made available by the authors, without undue reservation, to any qualified researcher.

## Ethics Statement

The studies involving human participants were reviewed and approved by the Ethics Committee of the National Rehabilitation Aids Research Center. The patients/participants provided their written informed consent to participate in this study.

## Author Contributions

ZLi designed the study and edited the manuscript. YL did the experiment, analyzed the data, and drafted the manuscript. KL and QL did the experiment. GX analyzed the data. CH performed the statistical analysis. RJ, TZ, and PS contributed to the physiological interpretation of the results. ZLv edited the manuscript. All authors contributed to the article and approved the submitted version.

## Conflict of Interest

The authors declare that the research was conducted in the absence of any commercial or financial relationships that could be construed as a potential conflict of interest.

## Appendix

### Calculation Formula of Gait Symmetry Indices

1. The phase symmetry index (IDps) (Yang et al., 2001; Wang et al., 2005; Jin and Zhang, 2011; Gouwanda, 2014; Wu and Pan, 2014) is calculated to evaluate the symmetry of the left and right leg gaits by the time phase of the gait cycle.

(1) $$\text{IDps}=\sqrt{\frac{T_{0}}{T}}\,\left(0.62\,\frac{S_{\text{s}}}{s_{\text{m}}}+0.38\,\frac{W_{s}}{W_{m}}\right),$$

where *T*_0_ is the average of the gait cycle of healthy people; *T* is the gait cycle of the measured object; *S*_s_ and *S*_m_ are the minimum and maximum values, respectively, of the single leg support period; and *W*_s_ and *W*_m_ are the minimum and maximum values, respectively, of the single leg swing period. The different gait cycles of the left and right legs are divided into the left relative symmetry index (IDpsL) and the right relative symmetry index (IDpsR).

2. The F-step size symmetry index in the Cartesian coordinate system, which is a comprehensive symmetry index based on the Fitts’ law (Yang et al., 2001; Wang et al., 2005; Jin and Zhang, 2011; Gouwanda, 2014; Wu and Pan, 2014), is calculated as follows:

(2) $$\text{IDsp}=\frac{l\,o\,g_{2}\,\left(2\,S\,T_{1}/S\,T_{l\,0}\right)/t_{1}}{l\,o\,g_{2}\,\left(2\,S\,T_{r}/S\,T_{r\,0}\right)/t_{r}},$$

where *ST*_1_ and *ST*_r_ are the stride lengths of the left and right legs, respectively; *ST*_*l0*_ and *ST*_r0_ are the step by step precision error values of the left and right legs, respectively, of a healthy person with a normal gait; and *t*_1_ and *t*_r_ are the swing period of the left and right legs, respectively.

3. The step size symmetry index (Yang et al., 2001; Wang et al., 2005; Jin and Zhang, 2011; Gouwanda, 2014; Wu and Pan, 2014) in polar coordinates (IDsp*θ* and IDsp*ρ*) is calculated using the following equations:

(3) $$\text{IDsp}\,\theta =\frac{l\,o\,g_{2}\,\left(2\,\theta_{S\,T_{s}}/\theta_{S\,T_{0}}\right)/t_{s}}{l\,o\,g_{2}\,\left(2\,\theta_{S\,T_{m}}/\theta_{S\,T_{0}}\right)/t_{m}}\,\text{and}$$

(4) $$\text{IDsp}\,\rho =\frac{l\,o\,g_{2}\,\left(2\,\rho_{S\,T_{s}}/\rho_{S\,T_{0}}\right)/t_{s}}{l\,o\,g_{2}\,\left(2\,\rho_{S\,T_{m}}/\rho_{S\,T_{0}}\right)/t_{m}},$$

where *θ* and *ρ* are the absolute values of the difference between the polar angle and the polar diameter of the toe during motion and at the end of the motion relative to the hip joint, respectively; *θ_*ST*_*_s_ and *ρ_*ST*_*_s_ are the smaller values of the spans; *θ_*ST*_*_m_ and *ρ_*ST*_*_m_ are the larger values among the stride lengths; *t*_s_ and *t*_m_ are the smaller and larger side step execution times, respectively, of log_2_(2*θ_*ST*_*/*θ_*ST*_*_0_)/*t*; and *θ_*ST*_*_0_ and *ρ_*ST*_*_0_ are the precision error values of the normal step size of the normal person.

4. The asymmetry index (ASI) (Yang et al., 2001; Wang et al., 2005; Jin and Zhang, 2011; Gouwanda, 2014; Kutilek et al., 2014; Wu and Pan, 2014) is calculated using the following equation:

(5) $$\text{ASI}=\left|\frac{2\,\left(X_{\text{L}}-X_{\text{R}}\right)}{X_{\text{L}}+X_{\text{R}}}\right|\times 100\%,$$

where *X*_L_ and *X*_R_ are the support times of the single support phase of the left and right feet, respectively.

### Formula of WPCO

In the calculation, if the original signal undergoes wavelet transform and the instantaneous wavelet phase is at a certain frequency *f* and time *t*_n_ as (Yang et al., 2001)

(6) $$\emptyset_{k}\,\left(f,t_{n}\right)=\text{arctan}\,\left[\frac{b_{k}\,\left(f,t_{n}\right)}{a_{k}\,\left(f,t_{n}\right)}\right],$$

then the instantaneous phase difference between these two signals is

(7) $$△\,\emptyset \,\left(f,t_{n}\right)=\emptyset_{1}\,\left(f,t_{n}\right)-\emptyset_{2}\,\left(f,t_{n}\right).$$

Averaging cos△∅(*f*,*t*_*n*_) and sin△∅(*f*,*t*_*n*_) in the time domain, the following is obtained:

(8) $$<\text{cos}\,△\,\emptyset \,\left(f\right)\geq \frac{1}{N}\,\sum_{n-1}^{N}\text{cos}\,△\,\emptyset \,\left(f,t_{n}\right)$$

(9) $$<\text{sin}\,△\,\emptyset \,\left(f\right)\geq \frac{1}{N}\,\sum_{n-1}^{N}\text{sin}\,△\,\emptyset \,\left(f,t_{n}\right)$$

Therefore, the definition of WPCO is

(10) $$C_{\phi }\,\left(f\right)=\sqrt{{\left\langle c\,o\,s\,\phi \,\left(f,t\right)\right\rangle }^{2}+{\left\langle s\,i\,n\,\Delta \,\phi \,\left(f\right)\right\rangle }^{2}}.$$
